# Traditional and advanced electrocardiographic measures of autonomic function in the population-based KORA-F3 study

**DOI:** 10.1007/s10654-025-01248-3

**Published:** 2025-06-26

**Authors:** Aenne S. von Falkenhausen, Felix N. Wenner, Luisa Freyer, Lauren E. Sams, Margit Heier, Annette Peters, Birgit Linkohr, Steffen Massberg, Axel Bauer, Stefan Kääb, Konstantinos D. Rizas, Moritz F. Sinner

**Affiliations:** 1 Department of Cardiology, LMU University Hospital Munich, Ziemssenstr. 5, 80336 Munich, Germany; 2 German Centre for Cardiovascular Research (DZHK), partner site: Munich Heart Alliance, Munich, Germany; 3 Institute of Epidemiology, Helmholtz Zentrum München, German Research Center for Environmental Health GmbH, Neuherberg, Munich, Germany; 4 KORA Study Centre, University Hospital of Augsburg, Augsburg, Germany; 5 Institute for Medical Information Processing, Biometry and Epidemiology (IBE), Faculty of Medicine, LMU Munich, Munich, Germany; 6 German Centre for Diabetes Research (DZD), Partner Site: Munich-Neuherberg, Munich-Neuherberg, Germany; 7 University Hospital for Internal Medicine III, Department of cardiology and angiology, Medical University Innsbruck, Innsbruck, Austria

**Keywords:** Heart-rate variability, Periodic repolarization dynamics, Deceleration capacity, Autonomic tone, ECG

## Abstract

**Aims:**

Heart-rate variability (HRV) measures are surrogates of autonomic function at the level of the sinus node and have evolved as markers of cardiovascular mortality in patients after myocardial infarction (MI). Traditionally, HRV is assessed in time-domain and frequency domain. Advanced measures of autonomic function include deceleration capacity (DC) and periodic repolarization dynamics (PRD). DC predominantly quantifies the influence of parasympathetic tone. PRD captures low-frequency oscillations of repolarization instability and is considered to reflect sympathetic activity at the level of the left ventricular myocardium. However, population-based reference values are missing.

**Methods and results:**

In 505 participants of the population-based KORA F3 study (Cooperative Health Research in the Region of Augsburg) with extant digital 24-h Holter electrocardiograms we assessed markers of HRV in time and frequency domains. Additionally, we determined advanced measures of autonomic function including DC and PRD applying previously established technologies. We used standard, pre-defined cut-off values to define high-risk groups. The cohort’s mean age was 63.6 ± 5.5 years, and 256 (50.1%) were women. Among HRV measures, exemplarily the median standard deviation of all normal-to-normal intervals (SDNN) was 141 ms [119;165] and the median low frequency to high frequency ratio (LF/HF-ratio) was 3.92 [2.69;6.18]. Regarding autonomic function, median DC was 5.32 ms [2.69;6.18], and median PRD was 2.92 ms [2.06;4.14]. Among these measures LF/HF-ratio was significantly higher among men (5.15 [3.23; 7.20]) than women (3.37 [2.36;4.53], *p* < 0.001). Measured distribution is also provided in a cohort subset without overt cardiovascular conditions. While DC decreased with age, SDNN, LF/HF-ratio, and PRD were stable across age-groups. For participants with comorbidities including hypertension, intake of betablockers, history of MI, stroke, or diabetes mellitus significantly lower SDNN, LF/HF-ratio, and DC were observed.

**Conclusion:**

In a large population-based cohort, we systematically present traditional and advanced measures of HRV of cardiac autonomic function. We report reference values in the overall cohort, as well as stratified by sex, age, and concomitant cardiovascular conditions.

## Introduction

The autonomous nervous system (ANS) and its influence on cardiac function are of great physiological and pathophysiological interest [1]. Both sympathetic and parasympathetic tone modulate inotropic, chronotropic, dromotropic, and bathmotropic effects on the heart. Autonomic tone thereby confers complex interaction to individually adapt cardiac function to varying circumstances [2, 3]. Conversely, inappropriately altered autonomic tone, particularly increased sympathetic tone and decreased vagal tone, is associated with elevated cardiovascular mortality. Underlying mechanisms include increased arrhythmogenicity, myocardial injury through oxygenic stress, and a detrimental influence promoting cardiovascular risk factors. For specific conditions like myocardial infarction (MI), particularly in the context of reduced ejection fraction, therapeutic interventions like betablocker therapy may attenuate the excess autonomic tone and thereby reduce cardiovascular mortality [4–9].

Whereas the relation of autonomic tone and cardiovascular outcome is established, it remains unclear how to best quantify an individual’s autonomic function. Heart rate can be measured non-invasively by electrocardiogram (ECG) and is immediately influenced by autonomic tone. Elevated heart rate has been linked to increased cardiovascular mortality [10, 11]. Heart rate variability (HRV) quantifies short- and long- term modulations of heart rate, which are primarily modulated by the ANS [12].

HRV is quantified by time-domain measures and frequency-domain measures. In addition, more advanced markers of cardiac autonomic function exist. Deceleration capacity (DC), a derivate of HRV, uses the integral of the sum of all RR-intervals during a heart rate slowdown and predominantly quantifies the influence of vagal tone on the sinoatrial node [13]. Periodic repolarization dynamics (PRD), a marker of repolarization instability, dynamically assesses periodic changes of the T-wave at the level of the ventricular myocardium, which is predominantly controlled by the sympathetic nervous system. Thus, PRD is considered to be a marker of cardiac sympathetic tone [14]. Most measures have been validated prospectively, and cut-off values for SDNN, DC, and PRD have been found to be associated with increased cardiovascular mortality after MI, as well as in the context of other cardiovascular conditions [15–22].

Despite the wide-spread use, to date no population-based reference values for most of these measures exist. Here, we use the population-based KORA Study to systematically describe measures capturing important aspects of HRV and advanced measures of cardiac autonomic function, including their age- and sex-specific distributions, and to establish population-based reference values.

## Methods

### Study cohort

The population-based KORA study (Cooperative Health Research in the Region of Augsburg) started in 1984 as one of the study regions of the World Health Organization MONICA project (Monitoring of trends and determinants in cardiovascular disease) [23–26]. In 1994/95, the S3 survey enrolled 4,856 (conversion rate 74.9%) men and women of German nationality between 25 and 74 years of age, randomly selected through the registration office in the Augsburg Region in Southern Germany. In 2004/05, the KORA F3 study was performed as a 10-year follow-up of KORA S3, and 3,006 still available individuals (conversion rate 75.9%) agreed to a participate. Of these, 546 participants 54 to 79 years of age were selected maintaining the original age- and sex-distribution of KORA F3. In this subgroup of KORA F3, digital 12-lead 24-h Holter-ECGs are available, and they constitute the study cohort. For all participants, detailed demographic, anthropometric, medical history, and physical examination data were recorded, as reported before [23]. All participants provided written informed consent, and the study methods were approved by the responsible ethics committee of the Bavarian Chamber of Physicians (EK No. 03097).

### Definition of comorbidities

Hypertension was defined as a systolic blood pressure ≥ 140 mmHg, a diastolic blood pressure ≥ 90 mmHg in accordance with the ESC guidelines for the management of elevated blood pressure and hypertension [27], or the intake of antihypertensive medication, when known to be taken for high blood pressure. Diabetes was considered prevalent by participant self-report and also diagnosed upon intake of antidiabetic medication. The prevalence of MI, stroke, and diabetes mellitus was adjudicated by participant self-report. Medication use in the preceding seven days was validated by trained study personnel based on anatomical therapeutic chemical classification (ATC) codes.

### Electrocardiogram analysis

In the study cohort, digital 12-lead 24-h Holter-ECGs were recorded using a SEER MC digital 12-channel Holter monitoring system (*General Electric, Chicago, IL, USA*) with a sampling rate of 500 Hz. Recorders were applied by trained study technicians adhering to a standardized protocol. Upon completion, recordings were stored digitally until further use.

ECG analysis was performed by specifically trained investigators blinded to any other participant data. ECG data obtained from the recorders were re-formatted into the open ISHNE-format (International Society for Holter and Noninvasive Electrocardiology) and subsequently converted to an orthogonal Frank-lead configuration applying the inverse Dower-transformation, as described before [28, 29]. A customized, previously validated [20, 30] Matlab-based software (SMARTlab v 1.54) was used for ECG analysis. We report heart rate and corrected QT interval (QTc) according to Bazett’s formula. All HRV measures in time and frequency domain, as well as the advanced indices of cardiac autonomic function were calculated according to the recommendations provided by the Task Force of The European Society of Cardiology and the North American Society for Pacing and Electrophysiology [12]. In brief, all ECGs underwent rigorous quality control. Artefact-rich sections were marked and excluded. We also excluded ECGs exhibiting atrial fibrillation, atrial flutter, or pacemaker stimulation. For the analysis of conventional HRV measures and DC, we used the entire ECG recording. For PRD analysis we selected 6-h segments between 9 pm and 3am, as previously described [12]. The final analysis dataset encompassed 505 quality-controlled ECGs with complete data for all ECG measures.

For traditional HRV-measures, we present time domain measures (SDNN—Standard Deviation of the Normal-to-Normal interval; RMSSD—Root Mean Square of Successive Differences between normal heartbeats; HRVi—HRV triangular index) and frequency domain measures (LF—Low Frequency; HF—High Frequency; LF/HF-ratio; ULF—Ultra low frequency; VLF—Very low frequency) and the advanced markers of cardiac autonomic function, DC and PRD. The calculation of these measures was performed according to the recommendations of the Task Force of the European Society of Cardiology and the North American Society of Pacing and Electrophysiology (12). DC was calculated by application of the Phase-Rectified Signal Averaging (PRSA) technique, as previously described. The PRSA-signal can be considered as a condensed version of the original RR-interval time series, including all periodic components of HRV related to decelerations. The central part of the PRSA-signal is quantified by wavelet-analysis to obtain the numerical measure of DC [13, 31, 32].

PRD was calculated as previously reported [14, 33]. In brief, T-wave vectors were derived from three-dimensional Frank leads and were used to determine the angle between two successive T-waves (*dT°*). Temporal integration of *dT°* using wavelet transformation unravels low-frequency (< 0.1 Hz) periodic components of repolarization instability.

The specific algorithms to determine DC and PRD can be accessed via the following link (https://osf.io/jz3w2/).

## Statistical analysis

We describe cohort characteristics in the overall cohort, as well as stratified by sex. We further describe Holter-ECG measures overall and stratified by sex and by age group. In addition, we present results in a restricted subset of the overall cohort limited to those without overt cardiovascular conditions defined as myocardial infarction, diabetes, or stroke, overt atrial fibrillation or QRS > 120 ms (“apparently healthy”). The distribution range between the 1st and the 99th percentile was used to define reference values. Discrete data are presented as absolute and relative frequencies and are compared using Fisher’s exact tests. Continuous data are presented as means ± standard deviation and are compared by Student t-tests or as medians with 25th-75th percentiles and are compared by Wilcoxon tests. The correlation between Holter-ECG measures with age is tested using Pearson’s or Spearman’s correlation, as appropriate. Pearson’s correlation is further used to assess correlation between ECG measures.

We plot the distribution of traditional and advanced measures using histograms. We further fit linear (SDNN, DC) and median (PRD, LF/HF) regression models to account the respective measures’ distributions for age. The results are visualized by scatter plots and the predicted regression lines including 95% confidence interval bands.

For selected ECG measures, clinical cut-off points have been described: For SDNN ≤ 100 ms vs. > 100 ms [11]; for DC ≤ 2.5 ms vs. > 2.5 ms [13, 16]; for PRD < 5.75deg^2^ vs. ≥ 5.75deg^2^ [14, 33]. For LF/HF-ratio no consistent cut-off has been reported. We thus use the median as the separating value. We plot the absolute and relative frequencies of these cut-offs in our cohort. We finally define relevant clinical conditions and compare the propensity of individuals exceeding the cut-offs of the selected ECG measures across these conditions. Conditions include (a) sex; (b) age groups; (c) the presence or absence of arterial hypertension; (d) the use (yes or no) of betablockers; (e) the presence or absence of any combination of myocardial infarction, stroke, or diabetes mellitus.

Statistical analyses were performed using R (version 4.3.1, The R Foundation for Statistical Computing, Vienna, Austria) embedded in R-Studio (version 1.2.1335, Integrated Development for R, RStudio, Boston, MA). We consider a two-sided *p* < 0.05 significant.

## Results

Following exclusions and quality control, 505 KORA F3 participants with analyzable 24-h Holter ECGs were available. Their mean age was 63.6 ± 5.5 years, and 256 (50.1%) were females. Cohort characteristics are described in Table 1. Cardiovascular risk factors were prevalent: 64.4% showed hypertension, 9.5% had a diagnosis of diabetes mellitus or used antidiabetic medication, 2.4% presented with a prior MI, and 23.0% were prescribed betablockers. Overall, men and women exhibited similar baseline characteristics. Whereas the percentage of those with hypertension was similar, the measured systolic and diastolic blood pressure was better controlled in women. Women also used betablockers more frequently.

**Table 1:** Cohort characteristics

	Overall n = 505	Women n = 256	Men n = 249	*p*
Age [years]	63.6 ± 5.5	63.4 ± 5.4	63.7 ± 5.5	0.51
BMI [kg/m^2^]	28.1 ± 4.0	27.9 ± 4.3	28.3 ± 3.7	0.29
Systolic blood pressure, [mmHg]	136.0 ± 26.0	133.5 ± 28.0	138.0 ± 23.0	< 0.001*
Diastolic blood pressure, [mmHg]	83.5 ± 14.1	82.0 ± 12.5	86.5 ± 14.0	< 0.001*
Active smoking, n (%)	60 (11.9%)	31 (12.1%)	29 (11.6%)	0.89
*Concomitant conditions*				
Diabetes mellitus, n (%)	48 (9.5%)	21 (8.2%)	27 (10.8%)	0.36
Hypertension, n (%)	325 (64.4%)	155 (60.5%)	170 (68.3%)	0.08
Myocardial infarction, n (%)	12 (2.4%)	5 (2.0%)	7 (2.8%)	0.57
Stroke, n (%)	16 (3.2%)	6 (2.3%)	10 (4.0%)	0.32
GFR, [ml/min]	81.3 ± 14.3	80.7 ± 15.0	81.9 ± 13.6	0.34
Renal Insufficiency (GFR < 30 ml/min), n (%)	2 (0.4%)	0 (0%)	2 (0.8%)	0.24
Coronary Revascularization, n (%)	12 (2.4%)	5 (2.0%)	7 (2.8%)	0.57
*Concomitant medication*				
Beta blockers, n (%)	116 (23.0%)	70 (27.3%)	46 (18.5%)	0.020*
Statins, n (%)	71 (14.1%)	36 (14.1%)	35 (14.1%)	1.00
ACE inhibitor, n (%)	80 (15.8%)	38 (14.8%)	42 (16.9%)	0.54
ARB, n (%)	44 (8.7%)	19 (7.4%)	25 (10.0%)	0.34
Diuretics, n (%)	99 (19.6%)	50 (19.5%)	49 (19.7%)	1.00
Antiplatelet therapy, n (%)	73 (14.5%)	33 (12.9%)	40 (16.1%)	0.31
Anticoagulation, n (%)	3 (0.6%)	0 (0%)	3 (1.2%)	0.12

Data expressed as mean ± standard deviation and as absolute and relative frequencies as appropriate. BMI: Body mass index; ACE: angiotensin converting enzyme, ARB: angiotensin II receptor blocker, GFR: Glomerular filtration rate

### Distribution of traditional HRV measures and advanced measures of cardiac autonomic function

**Fig. 1:**
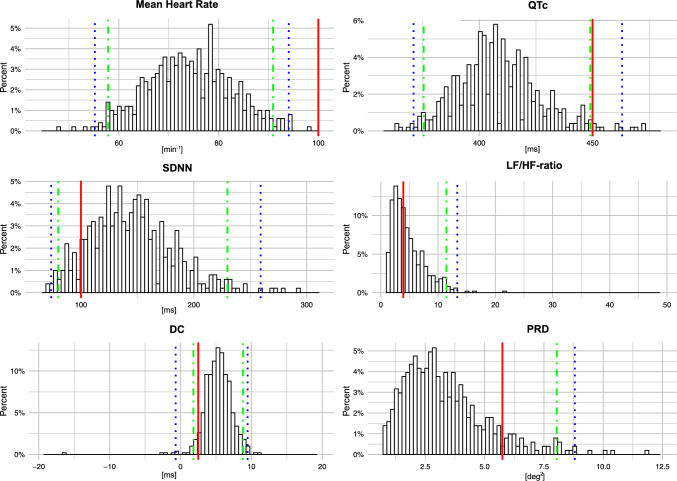
Distribution of traditional HRV measures and of advanced markers of cardiac autonomic function. Histogram distribution of HRV-measures in our cohort. Red lines showing clinically established cut-offs. Green lines representing 5% and 95% percentile, blue lines representing 1% and 99% percentile where appropriate. Abbreviations: SDNN—Standard Deviation of the Normal-to-Normal interval; LF—Low Frequency; HF—High Frequency ratio; DC—Deceleration Capacity; PRD—Periodic Repolarization Dynamics

The distribution and reference ranges of time-domain measures, of frequency-domain measures, as well as of the measures of cardiac autonomic function, stratified by sex, are presented in Table 2 for both the unstratified cohort and the “apparently healthy” subset. In the unstratified cohort, the mean heart rate and mean QTc interval (Bazett) were significantly higher in women than men (75 ± 8.7 vs. 73 ± 9.1 bpm, p = 0.011 and 412 ± 18.4 vs. 406 ± 19.5 ms, p = 0.001, respectively). Exemplarily for HRV measures and for advanced measures of cardiac autonomic function, we report a mean SDNN of 145 ± 38.5 ms (reference range 74–259 ms; men: 143 ± 41.2 ms vs. women: 146 ± 35.7 ms, *p* = 0.40). DC had a mean value of 5.25 ± 2.4 ms (reference range − 0.68–9.48 ms), which was not significantly different in men and women (5.25 ± 2.2 ms vs. 5.25 ± 2.6 ms, *p* = 0.98). PRD had a median value of 2.92deg^2^ [2.06;4.14] (reference range 0.82–8.81deg^2^), without significant difference between males (2.91deg^2^ [2.15;4.32]) and females (2.97deg^2^ [1.98;4.02], *p* = 0.43). Distribution details are provided in Table 2 and illustrated in Fig. 1.

**Table 2:** Distribution of traditional HRV measures and advanced measures of cardiac autonomic function

		Unstratified				Apparently healthy			
ECG measure		All n = 505	Women n = 256	Men n = 249	*p*	All n = 428	Women n = 225	Men n = 203	p
Heart rate [min^−1^]	mean ± SD	74.0 ± 8.7	75.0 ± 8.2	73.0 ± 9.1	0.011*	74.0 ± 8.6	74.9 ± 8.3	72.9 ± 8.9	0.017*
	1% Percentile	55.2	57.7	52.2	0.016*	55.5	57.7	53.5	0.025*
	5% Percentile	59.7	60.6	58.8		59.8	60.5	59.3	
	25% Percentile	68.3	69.4	66.8		68.2	69.3	66.8	
	50% Percentile	73.8	75.1	72.6		73.9	75.1	72.6	
	75% Percentile	79.9	80.0	79.8		79.6	79.9	79.6	
	95% Percentile	88.5	89.5	87.2		88.5	89.5	87.1	
	99% Percentile	94.1	94.3	92.7		94.0	94.2	92.4	
QTc [ms]	mean ± SD	409 ± 19.1	412 ± 18.4	406 ± 19.5	0.001*	408 ± 18.6	411 ± 18.1	405 ± 18.7	0.002*
	1% Percentile	371	369	372	< 0.001*	370	367	371	< 0.001*
	5% Percentile	381	385	377		379	384	376	
	25% Percentile	396	400	392		396	400	392	
	50% Percentile	407	410	404		407	410	404	
	75% Percentile	420	422	418		420	421	418	
	95% Percentile	444	445	437		439	445	435	
	99% Percentile	463	462	465		459	459	453	
*Time* *domain* *measures*									
SDNN [ms]	mean ± SD	145 ± 38.5	146 ± 35.7	143 ± 41.2	0.40	146 ± 38.7	148 ± 35.7	145 ± 41.8	0.50
	1% Percentile	74	72	75	0.17	78	77	78	0.19
	5% Percentile	87	92	85		89	93	88	
	25% Percentile	119	124	113		120	124	112	
	50% Percentile	141	143	139		144	145	141	
	75% Percentile	165	166	164		167	170	165	
	95% Percentile	216	204	218		218	206	220	
	99% Percentile	259	242	267		266	244	274	
RMSSD [ms]	mean ± SD	21.8 ± 8.7	22.4 ± 8.5	21.4 ± 8.8	0.12	21.8 ± 8.7	22.4 ± 8.5	21.2 ± 8.9	0.13
	1% Percentile	8.2	9.5	8.1	0.06	8.3	10.1	8.2	0.06
	5% Percentile	10.9	11.3	10.3		10.8	11.2	10.2	
	25% Percentile	15.8	16.5	14.9		15.9	16.5	14.9	
	50% Percentile	20.5	20.8	20.0		20.4	20.8	19.7	
	75% Percentile	26.4	26.5	26.3		26.3	26.3	25.7	
	95% Percentile	37.6	39.3	35.7		37.8	39.4	34.6	
	99% Percentile	45.8	45.2	45.3		45.9	45.5	45.8	
HRVi	mean ± SD	71.0 ± 22.6	70.5 ± 20.5	71.5 ± 24.7	0.62	71.9 ± 22.3	71.1 ± 20.5	72.8 ± 24.2	0.44
	1% Percentile	28.1	27.4	28.5	0.92	28.6	28.8	29.7	0.67
	5% Percentile	37.6	39.4	35.6		39.4	41.7	38.8	
	25% Percentile	55.7	56.9	53.4		56.9	57.3	56.5	
	50% Percentile	68.9	67.6	69.3		69.1	67.8	69.4	
	75% Percentile	83.8	83.5	84.5		84.8	83.8	85.3	
	95% Percentile	113.1	107.7	114.6		113.1	110.2	113.8	
	99% Percentile	135.3	122.8	146.6		135.2	123.5	151.6	
*Frequency* *domain* *measure*s									
LF [ms^2^]	mean ± SD	1,039 ± 1,675	1,086 ± 1,728	991 ± 1,622	0.52	1,044 ± 1,570	1,118 ± 1,764	961 ± 1,319	0.30
	1% Percentile	63	61	66	0.75	74	83	69	0.61
	5% Percentile	124	140	115		135	148	133	
	25% Percentile	284	280	296		300	292	311	
	50% Percentile	469	456	473		487	475	501	
	75% Percentile	922	905	947		948	920	991	
	95% Percentile	3,842	4,269	3,345		3,823	4,473	3,211	
	99% Percentile	8,609	8,671	8,308		8,366	8,711	7,702	
HF [ms^2^]	mean ± SD	260 ± 499	283 ± 493	236 ± 505	0.29	262 ± 459	296 ± 520	223 ± 377	0.10
	1% Percentile	11	19	11	< 0.001*	12	21	11	< 0.001*
	5% Percentile	26	34	22		29	34	23	
	25% Percentile	66	90	54		67	90	54	
	50% Percentile	132	148	100		130	147	98	
	75% Percentile	245	291	212		257	304	217	
	95% Percentile	844	805	966		895	822	974	
	99% Percentile	2,529	2,565	2,178		2,522	2,603	1,760	
LF/HF-ratio	mean ± SD	4.84 ± 3.46	3.96 ± 2.31	5.76 ± 4.15	< 0.001*	4.95 ± 3.57	3.98 ± 2.23	6.03 ± 4.39	< 0.001*
	1% Percentile	1.34	1.16	1.42	< 0.001*	1.35	1.24	1.58	< 0.001*
	5% Percentile	1.63	1.46	1.87		1.73	1.64	1.93	
	25% Percentile	2.69	2.36	3.23		2.78	2.42	3.49	
	50% Percentile	3.92	3.37	5.15		4.07	3.42	5.35	
	75% Percentile	6.18	4.53	7.20		6.42	4.52	7.40	
	95% Percentile	10.71	8.43	11.29		10.78	8.48	11.50	
	99% Percentile	11.62	11.14	15.07		13.26	11.01	16.51	
ULF [ms^2^]	mean ± SD	9,701 ± 6,740	9,966 ± 6,200	9,428 ± 7,257	0.37	9,936 ± 6,988	10,087 ± 6,363	9,768 ± 7,637	0.64
	1% Percentile	1,821	1,682	2,071	0.03*	1,827	1,593	2,178	0.09
	5% Percentile	2,950	3,200	2,733		2,993	3,004	3,036	
	25% Percentile	5,333	5,613	4,983		5,381	5,634	5,236	
	50% Percentile	7,788	8,247	7,574		7,846	8,315	7,586	
	75% Percentile	12,154	12,896	10,526		12,619	12,843	11,300	
	95% Percentile	23,866	23,136	24,505		24,492	23,780	24,747	
	99% Percentile	33,575	31,311	37,067		34,001	32,346	39,505	
VLF [ms^2^]	mean ± SD	2456 ± 4882	2,673 ± 5,369	2,232 ± 4,323	0.31	2,427 ± 4,596	2,627 ± 4,967	2,204 ± 4,144	0.34
	1% Percentile	204	182	290	0.54	267	204	323	0.40
	5% Percentile	408	421	406		431	445	412	
	25% Percentile	757	785	730		810	806	849	
	50% Percentile	1,215	1,179	1,265		1,235	1,198	1,278	
	75% Percentile	1,935	1,927	1,966		2,031	1,921	2,081	
	95% Percentile	8,310	9,640	6,514		8,171	9,392	6,047	
	99% Percentile	30,346	30,251	24,785		28,650	28,650	14,889	
*Measures* *of* *Autonomic* *Tone*									
DC [ms]	mean ± SD	5.25 ± 2.4	5.25 ± 2.6	5.25 ± 2.2	0.98	5.39 ± 2.25	5.39 ± 2.28	5.40 ± 2.23	0.98
	1% Percentile	− 0.68	1.09	− 0.76	0.25	− 0.18	1.54	− 0.83	0.50
	5% Percentile	2.46	2.84	2.25		2.76	2.86	2.36	
	25% Percentile	4.12	4.36	3.92		4.28	4.39	4.08	
	50% Percentile	5.32	5.44	5.26		5.48	5.55	5.40	
	75% Percentile	6.44	6.35	6.45		6.51	6.49	6.51	
	95% Percentile	8.30	7.85	8.49		8.37	7.94	8.62	
	99% Percentile	9.48	9.03	9.97		9.46	9.07	9.48	
PRD [deg^2^]	mean ± SD	3.34 ± 1.8	3.37 ± 1.7	3.32 ± 1.9	0.75	3.28 ± 1.78	3.34 ± 1.74	3.21 ± 1.82	0.46
	1% Percentile	0.82	0.79	0.90	0.43	0.81	0.78	0.88	0.34
	5% Percentile	1.18	1.26	1.15		1.18	1.25	1.14	
	25% Percentile	2.06	2.15	1.98		2.02	2.12	1.92	
	50% Percentile	2.92	2.91	2.97		2.86	2.83	2.88	
	75% Percentile	4.14	4.31	4.02		4.07	4.21	3.88	
	95% Percentile	6.63	6.63	6.61		6.62	6.93	6.41	
	99% Percentile	8.81	8.29	10.26		8.79	8.32	10.13	

ECG measures in the overall cohort and stratified by sex. SDNN—Standard Deviation of the Normal-to-Normal interval; RMSSD—Root Mean Square of Successive Differences between normal heartbeats; HRVi—HRV triangular index; LF—Low Frequency; HF—High Frequency; ULF – Ultra low frequency; VLF—Very low frequency; DC—Deceleration Capacity; PRD—Periodic Repolarization Dynamics; Apparently healthy subjects are participants without history of either myocardial infarction, diabetes or stroke, overt atrial fibrillation or QRS > 120 ms

As for the unstratified cohort, distribution details of the “apparently healthy” subset are described in Table 2. In this subset, we had excluded individuals with common preconditions including those with a history of myocardial infarction, diabetes, or stroke, overt atrial fibrillation, or a QRS > 120 ms. Across both the unstratified and the “apparently healthy” groups, all ECG-derived parameters showed largely similar distributions, both overall and stratified by sex. Additionally, no relevant correlation was noted between selected measures (Table 3).

**Table 3:** Correlation between selected measures

	Mean HR	QTc	SDNN	LF/HF-ratio	DC	PRD
Mean HR	**1**	− 0.06 (− 0.15;0.03) *p* = 0.19	− 0.43 (− 0.50;− 0.36) *p* < 0.001*	0.02 (− 0.07;0.11) *p* = 0.66	− 0.28 (− 0.36;− 0.20) *p* < 0.001*	0.24 (0.16;0.32) *p* < 0.001*
QTc	− 0.06 (− 0.15;0.03) *p* = 0.19	1	0.05 (− 0.04;0.14) *p* = 0.28	− 0.03 (− 0.12;0.06) *p* = 0.54	− 0.04 (− 0.13;0.05) *p* = 0.39	0.10 (0.01;0.19) *p* = 0.03*
SDNN	− 0.43 (− 0.50;− 0.36) p < 0.001*	0.05 (− 0.04;0.14) p = 0.28	1	− 0.02 (− 0.11;0.07) p = 0.67	0.28 (0.20;0.36) p < 0.001*	− 0.16 (− 0.24;− 0.07) p < 0.001*
LF/HF-ratio	0.02 (− 0.07;0.11) *p* = 0.66	− 0.03 (− 0.12;0.06) *p* = 0.54	− 0.02 (− 0.11;0.07) *p* = 0.67	1	0.08 (− 0.003;0.17) *p* = 0.06	0.08 (− 0.009;0.17) *p* = 0.08
DC	− 0.28 (− 0.36;− 0.20) *p* < 0.001*	− 0.04 (− 0.13;0.05) *p* = 0.39	0.28 (0.20;0.36) *p* < 0.001*	0.08 (− 0.003;0.17) *p* = 0.06	1	− 0.13 (.0.21;− 0.04) *p* = 0.004*
PRD	0.24 (0.16;0.32) *p* < 0.001*	0.10 (0.01;0.19) *p* = 0.03*	− 0.16 (− 0.24;− 0.07) *p* < 0.001*	0.08 (− 0.009;0.17) *p* = 0.08	− 0.13 (.0.21;− 0.04) *p* = 0.004*	1

Correlation between HRV-measures. Correlation-coefficients with 95% confidence intervals in parentheses. HR—heart rate; SDNN—Standard Deviation of the Normal-to-Normal interval; LF—Low Frequency; HF—High Frequency; DC—Deceleration Capacity; PRD—Periodic Repolarization Dynamics

### Traditional HRV measures and advanced measures of cardiac autonomic function, age and concomitant conditions

**Fig. 2:**
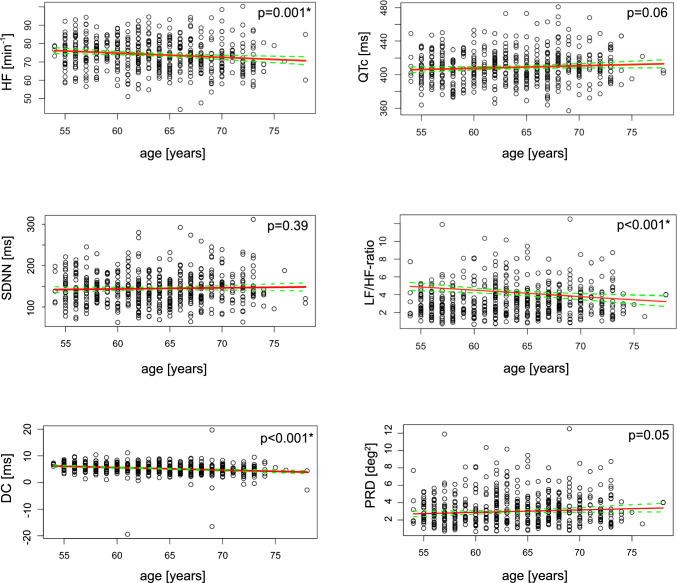
Characterization of the reported measures by age. Scatter plot distributions of Mean heart rate, QTc, SDNN, LF/HF-ratio, DC, and PRD including a predicted regression line for the relation with age. Abbreviations: SDNN—Standard Deviation of the Normal-to-Normal interval; LF—Low Frequency; HF—High Frequency; DC—Deceleration Capacity; PRD—Periodic Repolarization Dynamics

We investigated the change of HRV measures and of advanced measures of cardiac autonomic function with age in both the unstratified cohort and the “apparently healthy” subset. The mean or median distributions of each measure across different age-groups is provided in Table 4. In the unstratified cohort, mean heart rate and DC showed a significant decrease with increasing age. For selected measures, the relation with age is plotted together with a predicted regression line (Fig. 2).

**Table 4:** Traditional HRV measures and advanced measures of cardiac autonomic function in different age-groups

Parameter	cohort	all	age < 60	age ≥ 60, < 65	age ≥ 65, < 70	age ≥ 70	*p*	Correlation
Mean HR [min^−1^]	Unstratified	74.0 ± 8.7	75.5 ± 8.0	74.4 ± 8.9	73.2 ± 8.7	72.3 ± 9.2	0.029*	− 0.14 (− 0.23;− 0.06)
	„apparently healthy “	74.0 ± 8.6	75.6 ± 8.0	74.3 ± 8.8	73.1 ± 8.3	72.1 ± 9.4	0.033*	− 0.14 (− 0.24;− 0.05)
QTc [ms]	Unstratified	409 ± 19	406 ± 18	409 ± 18	410 ± 22	410 ± 18	0.30	0.08 (− 0.004;0.17)
	„apparently healthy “	408 ± 19	406 ± 18	408 ± 18	410 ± 21	408 ± 16	0.59	0.05 (− 0.04;0.14)
*Time* *domain* *measures*								
SDNN [ms]	Unstratified	145 ± 39	145 ± 35	141 ± 40	145 ± 40	149 ± 41	0.56	0.03 (− 0.06;0.12)
	„apparently healthy “	146 ± 39	146 ± 35	143 ± 39	148 ± 40	152 ± 43	0.44	0.05 (− 0.04;0.15)
RMSSD [ms]	Unstratified	21.8 ± 8.7	21.5 ± 7.7	21.5 ± 7.9	21.3 ± 8.5	23.9 ± 11.1	0.14	0.08 (− 0.01;0.16)
	„apparently healthy “	21.8 ± 8.7	21.4 ± 7.8	21.4 ± 7.7	21.6 ± 8.6	23.8 ± 11.6	0.26	0.08 (− 0.01;0.17)
HRVi	Unstratified	71.0 ± 22.6	73.6 ± 21.8	70.1 ± 23.9	69.0 ± 20.7	71.5 ± 25.0	0.38	− 0.04 (− 0.13;0.04)
	„apparently healthy “	71.9 ± 22.3	73.3 ± 21.4	71.8 ± 23.0	70.3 ± 20.2	72.6 ± 26.1	0.78	− 0.03 (− 0.12;0.07)
*Frequency* *domain* *measures*								
LF [ms^2^]	Unstratified	469 [284;922]	522 [312;986]	493 [305;981]	437 [251;794]	415 [275;871]	0.34	− 0.06 (− 0.14;0.03)
	„apparently healthy “	487 [300;948]	548 [333;997]	514 [312;989]	457 [274;790]	413 [277;910]	0.43	− 0.03 (− 0.13;0.06)
HF [ms^2^]	Unstratified	132 [66;245]	126 [73;227]	142 [73;258]	122 [54;219]	140 [65;280]	0.34	− 0.05 (− 0.14;0.04)
	„apparently healthy “	130 [67;257]	126 [75;239]	145 [77;258]	116 [55;240]	130 [64;350]	0.67	− 0.02 (− 0.12;0.07)
LF/HF− ratio	Unstratified	3.92 [2.69;6.18]	4.49 [3.08;7.30]	3.91 [2.46;6.20]	3.83 [2.81;5.75]	3.51 [2.36;4.89]	0.06	− 0.11 (− 0.20;− 0.03)
	„apparently healthy “	4.07 [2.78;6.42]	4.48 [3.12;7.33]	3.84 [2.64;6.40]	3.97 [2.88;6.01]	3.53 [2.43;4.93]	0.10	− 0.10 (− 0.19;− 0.005)
ULF [ms^2^]	Unstratified	7,788 [5,333;12,154]	8,326 [5,597;12,115]	6,907 [4,656;12,525]	7,655 [5,421;11,524]	7,773 [5,626;13,015]	0.42	0.02 (− 0.07;0.10)
	„apparently healthy “	7,846 [5,381;12,619]	8,405 [5,591;12,479]	6,946 [4,679;12,654]	7,861 [5,610;11,489]	7,846 [5,789;13,143]	0.48	0.03 (− 0.06;0.13)
VLF [ms^2^]	Unstratified	1,215 [757;8,310]	1,227 [855;2,169]	1,370 [731;1,969]	1,093 [685;1,694]	1,076 [750;1,815]	0.68	− 0.01 (− 0.10;0.08)
	„apparently healthy “	1,235 [810;2,031]	1,240 [867;2,204]	1,384 [763;2,101]	1,131 [794;1,677]	1,085 [779;1,764]	0.97	− 0.02 (− 0.11;0.08)
*Measures* *of* *Autonomic* *Tone*								
DC [ms]	Unstratified	5.3 ± 2.4	6.0 ± 1.6	5.3 ± 2.7	4.9 ± 2.8	4.6 ± 2.0	< 0.001*	− 0.22 (− 0.31;− 0.14)
	„apparently healthy “	5.4 ± 2.3	6.2 ± 1.6	5.4 ± 2.7	5.2 ± 2.3	4.5 ± 2.0	< 0.001*	− 0.25 (− 0.34;− 0.16)
PRD [deg^2^]	Unstratified	2.92 [2.06;4.14]	2.64 [1.95;3.79]	3.15 [2.13;4.60]	3.02 [2.07;4.12]	3.06 [2.09;4.32]	0.08	0.07 (− 0.02;0.16)
	„apparently healthy “	2.86 [2.02;4.07]	2.53 [1.85;3.63]	3.11 [2.12;4.39]	2.88 [1.94;4.03]	3.23 [2.17;4.39]	0.019*	0.12 (0.03;0.21)

Measures expressed as mean ± standard deviation or median [25th;75th percentile] as appropriate. HR—Heart rate; SDNN—Standard Deviation of the Normal-to-Normal interval; RMSSD—Root Mean Square of Successive Differences between normal heartbeats; HRVi—HRV triangular index; LF—Low Frequency; HF—High Frequency; ULF—Ultra low frequency; VLF—Very low frequency; DC—Deceleration Capacity; PRD—Periodic Repolarization Dynamics; “Apparently healthy” represents participants without history of either myocardial infarction, diabetes, or stroke, overt atrial fibrillation or QRS > 120 ms

For the “apparently healthy” subset, the distribution by age group is likewise presented in Table 4. As in the unstratified cohort, mean heart rate and DC declined with increasing age. In the “apparently health” subset, PRD significantly increased with increasing age, which was also observed, yet non-significantly, in the unstratified cohort.

**Fig. 3:**
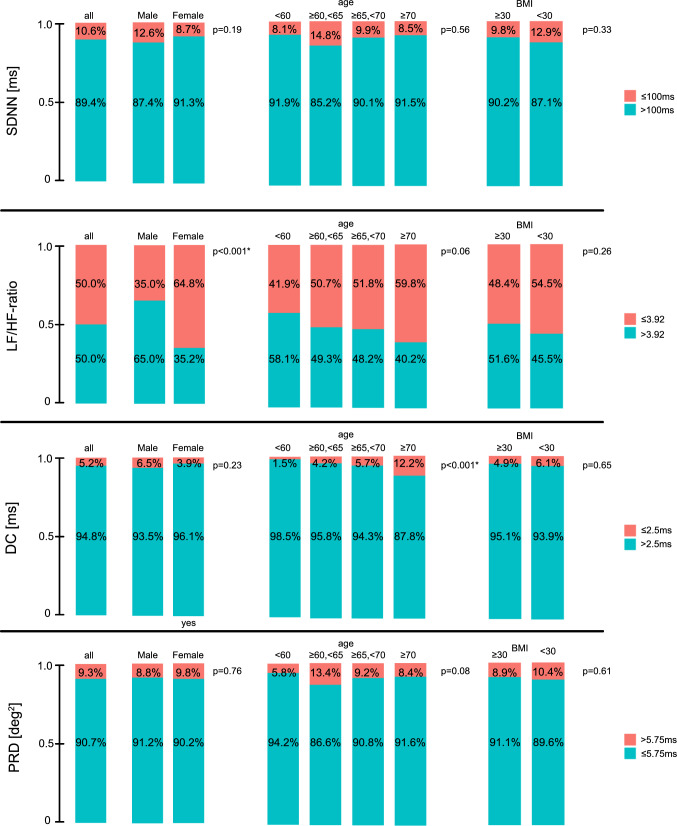
Propensity of clinically proposed cut-offs for the reported measures stratified by age and sex. For SDNN, DC, and PRD, relevant cut-off values have been proposed clinically. For LF/HF-ratio we used the median in lack of clinically established cut-off values. We present the propensity of these cut-off values for all parameters in our overall cohort (left panels). We also compare the propensity of these cut-off values in strata according to sex (second and third panels from the left), different age groups (mid panels) and Body mass index (BMI, right panel) with red illustrating the proportion of participants with potentially pathologic values. Abbreviations: SDNN—Standard Deviation of the Normal-to-Normal interval; LF—Low Frequency; HF – High Frequency; DC—Deceleration Capacity; PRD—Periodic Repolarization Dynamics; MI—Myocardial infarction

**Fig. 4:**
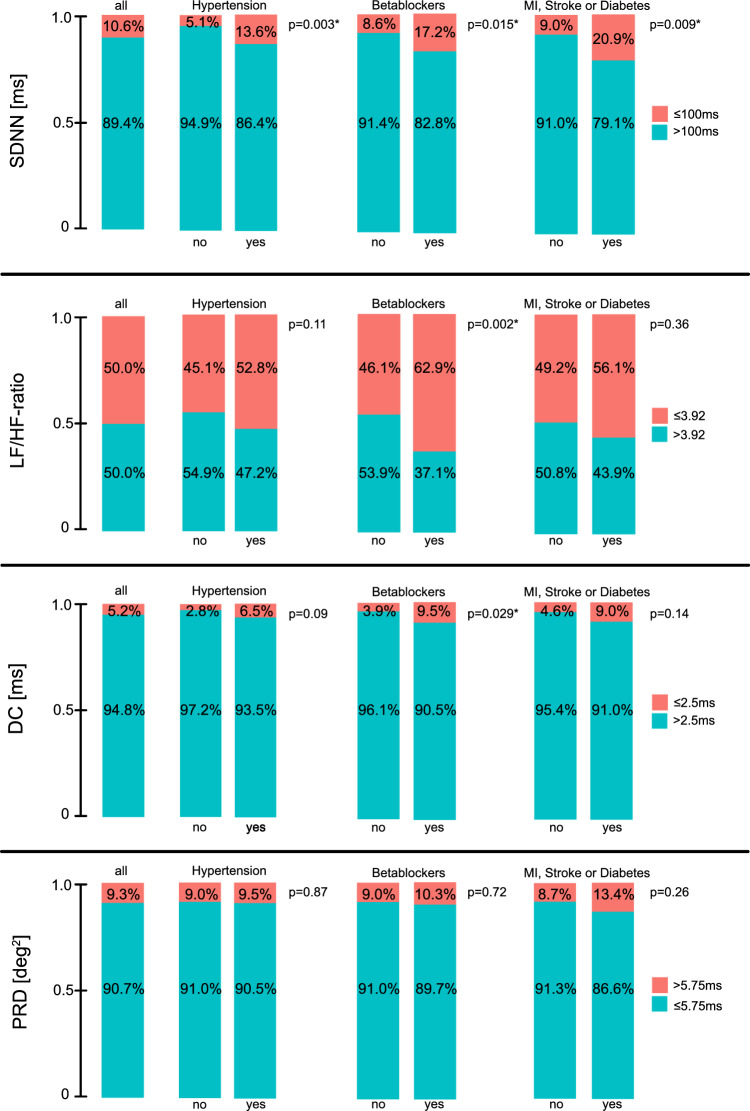
Propensity of clinically proposed cut-offs for the reported measures stratified by comorbidities. For SDNN, DC, and PRD, relevant cut-off values have been proposed clinically. For LF/HF-ratio we used the median in lack of clinically established cut-off values. We present the propensity of these clinical cut-off values for all parameters in our overall cohort (left panels). We also compare the propensity of these cut-off values in strata of our cohort (three right panels). The cohort was stratified by hypertension status (left), intake of betablockers (middle), and prevalence of concomitant diagnoses of myocardial infarction, stroke, or diabetes mellitus (right). Abbreviations: SDNN—Standard Deviation of the Normal-to-Normal interval; LF—Low Frequency; HF—High Frequency; DC—Deceleration Capacity; PRD—Periodic Repolarization Dynamics; MI—Myocardial infarction

Figures 3 and 4 illustrate the proportion of individuals exceeding clinically established cut-off values. Reflecting the population-based provenance of our cohort, around 9 out of 10 participants exhibited low risk values, where clinical cut-offs were available. We subsequently explored differences in the cut-off distributions across sex, age and body-mass index (BMI, Fig. 3). LF/HF-ratio showed a higher percentage of pathologic values in women compared to men (*p* < 0.001). All other selected measures revealed no significant differences between sexes. Of all measures, only DC showed a gradual increase in pathologic cut-off values with increasing age, ranging from 1.5 in participants age < 60 years to 12.2% in participants age ≥ 70 years (*p* < 0.001). When focusing on clinically relevant risk groups (Fig. 4), for SDNN and DC, the percentage of high-risk participants increased with the presence of hypertension (*p* = 0.003 and *p* = 0.09, respectively) and intake of betablockers (*p* = 0.015 and *p* = 0.029, respectively), and in case of SDNN, also with the prevalence of the concomitant conditions MI, stroke and diabetes mellitus (*p* = 0.009). There was no significant difference in the PRD distribution across these strata.

## Discussion

We report one of the first comprehensive descriptions of population-based reference values for traditional time-domain and frequency-domain HRV measures and advanced measures of cardiac autonomic function. Cumulatively, these measures capture different aspects of the ANS. Moreover, we describe the distribution of these measures across different sexes and age-groups.

The KORA Study is a population-based cohort, which was designed to represent the age- and sex-stratified population in the South German region around the city of Augsburg [23]. Balancing resources, in a subset of the KORA F3 survey digital 12-lead 24-h Holter-ECGs were conducted. This subset was confined to an age-spectrum from 54 to 79 years aiming to reflect the part of the population, which is prone to develop cardiovascular conditions. It was thus unclear how the reported measures in our cohort would compare to prior findings, particularly because many of these reports were derived from in-patient cohorts with major cardiovascular conditions [13–22]. HRV description in non-diseased cohorts are rare. One of the largest studies in such individuals enrolled healthy individuals only who were not representative for the general population [34]. Also, the methodology of this study and the interpretation of the results raised concerns [35].

The Task Force of the European Society of Cardiology and the North American Society of Pacing and Electrophysiology previously has proposed reference values for HRV measures. For the time-domain measure SDNN, the reference value is a mean of 141 ± 39 ms [12, 36]. Other sources report a mean of 166 ± 32 ms for SDNN [11, 18, 37–43]. Yet, many of these prior studies were small, partly relying on 14 healthy participants only [42]. In our study of over 500 participants, we report a mean SDNN of 145 ± 38.5 ms. Our findings are hence well in line with these previous reports.

For LF/HF-ratio as an example of a frequency domain measure of HRV, a relevant distribution difference by sex and minor distribution differences by age-group have been described [34, 43]. There, in the age-group > 50 years, which is closest to our current age distribution, the median LF/HF-ratio was 2.58 [1.64;3.57] in women and 3.08 [2.16;4.25] in men [34]. With a median LF/HF-ratio of 3.37 [2.36;4.53] in women and 5.15 [3.23;7.20] in men, values were markedly higher in our population-based study. The specific reasons for these differences remain incompletely understood. However, our analysis is based on a large, population-based cohort, whereas the respective prior values were based on relatively small subsets of volunteers.

Likewise for DC and PRD, population-based reference values are currently missing. For DC, we report a mean of 5.25 ± 2.4 ms in our unstratified cohort. DC has first been reported in 2006 in patients after an acute MI. In this initial description, the unstratified mean value was 5.6 ms [13]. For PRD, the population-based reference median in our study is 2.92 [2.06;4.14] deg^2^. Despite several reports on the clinical implications of PRD cut-offs [14, 19, 20, 22], the respective cohort median values had not been reported. Of note, the reported cut-off values for PRD and other measures were derived from diseased cohorts with a generally higher propensity of pathologic values. In our population-based cohort, concomitant conditions only occur with a population-based prevalence. Our cohort can thus be considered “healthier” and pathologic values occur less frequently.

The clinical applicability of measures depends on their stability in relevant strata of the population. Importantly, we have investigated differences in the distribution of traditional HRV measures and advanced measures of autonomic function across sex both in our unstratified cohort and an “apparently healthy” cohort subset without overt cardiovascular conditions. We observed some variability for the frequency-domain measure LF/HF-ratio, which is known to be affected by influencing factors including physiological, psychological, environmental, lifestyle, and genetic factors [44]. However, time-domain measures as well as the advanced marker DC and PRD, were distributed comparably in men and women, both in the unstratified cohort and in the “apparently healthy” subset. This is partly in contrast to a prior report suggesting these measures to be higher in men than in women [34]. Regarding age, our cohort covered individuals from 54 to 79 years. Time-domain and frequency-domain measures were quite stable across age-groups. However, DC constantly decreased with increasing age both in the unstratified cohort and in the “apparently healthy” subset. Whereas PRD appeared stable across age-groups in the unstratified cohort, in the “apparently healthy” subset a significant increase with increasing age was revealed. It has been suggested that a reduction of cardiac autonomic control during aging is particularly driven by a reduction in vagal tone leading to a predomination of sympathetic tone [45, 46]. Our results of an age-dependent decrease of parasympathetically mediated DC and reversely an age-dependent increase in sympathetically mediated PRD support these previous findings. Our “apparently healthy” cohort thus offer a slightly clearer view of physiologic aging, while the unstratified data may better reflect real-world clinical populations.

Cardiac autonomic tone is influenced by different ANS components. To examine, whether the selected measures tag independent aspects of autonomic tone, we systematically calculated correlations between them. We thereby noted that all parameters, especially DC and PRD, were not relevantly correlated, neither with other time- or frequency-domain measures nor among each other. We submit that this lack of correlation underpins the independent and incremental information provided by these measures.

Clinical interpretability of both HRV measures and of advanced measures of cardiac autonomic function is of great importance. It is critical if measure-specific cut-off values confer a quantifiable risk for outcomes and if the prevalence of such cut-off values is influenced by concomitant conditions. For the time-domain measure SDNN, a value ≤ 100 ms is predictive of sudden cardiac death (SCD) in patients following a MI [11]. Likewise, DC ≤ 2.5 ms has been associated with increased mortality in post-MI patients [13]. Finally, as a quantification of impaired sympathetic function, PRD ≥ 5.75 deg^2^ is the only measure that has been established as a SCD risk marker in patients with ischemic and non-ischemic cardiomyopathy [14, 21, 47]. However, such clinically established cut-off values derived from selected, pre-conditioned cohorts including post-MI cohorts might not be generalizable to a population-based cohort. Accordingly, for SDNN and PRD, only around 1 in 10 participants exhibited values exceeding and undercutting the clinically established cut-off values, respectively. Interestingly, for DC only 5.2% of participants fell below a DC cut-off of 2.5 ms. In our cohort it remains unclear if DC as a marker is more sensitive to subtle parasympathetic impairment, or if our results reflect that DC showed a significant age-dependency with lower values in older age-groups.

As mentioned, cut-off values for SDNN, DC, and PRD have been associated with increased mortality and partly (PRD) also for SCD [11, 13, 14]. Ideally, a predictor of SCD-risk should be independent of other influencing factors. To test the stability of our measure distributions in the context of possible influencing factors, we describe the propensity of cut-off values for SDNN, DC, and PRD depending on the presence of hypertension, MI, stroke, and diabetes mellitus, as well as the intake of betablockers, a commonly prescribed cardiovascular drug that is supposed to interact with the ANS. Given that no established cut-off for LF/HF-ratio has been published, we investigate the propensity of values below or above the overall cohort’s median. Both SDNN and DC were significantly modified by all three constellations, namely presence of hypertension, intake of betablockers or history of either stroke, MI or diabetes, with a higher propensity of pathologic values in the concomitant risk groups. Importantly, PRD was not significantly influenced by the three constellations, rendering it a very stable marker of cardiac autonomic function. This fact might be considered important for clinical applicability. Future research will need to investigate if SDNN, DC, and PRD, and eventually LF/HF-ratio, predict SCD and mortality more generally also in the general population rather than in specific subgroups that already are at an increased risk of events.

### Limitations

Some considerations are required when interpreting our data. First, our selected cohort is a subset KORA F3 study for which selection bias cannot fully be ruled out. However, this cohort is among the largest investigations to report reference values for ECG-based measures. The cohort is derived from the general population and consequently, it is not enriched for high-risk patients with severe cardiovascular conditions. Such conditions only occur at a population prevalence, which renders it difficult to investigate HRV measures in high-risk strata of the population. Also, the age range of our cohort was confined to 54 to 79 years. We hence cannot sufficiently adjudicate the generalizability to younger and older age groups. Similarly, the cohort comprises participants of German nationality. Although not suggested by prior data, HRV measures might be distributed differently in other geographic regions or in individuals of other descent. We report a comprehensive selection of traditional and advanced measures. However, numerous other measures exist which were not available in our cohort [48]. Methodologically, our measures were derived from standard 12-lead Holter ECGs recorded under ambulatory conditions with a resulting high activity level compared to severely diseased in-patients. A head-to-head comparison of measures across such very different underlying circumstances requires careful individual interpretation. It will be important to analyze if pathologic measures obtained on a population level can also be interpreted similarly to those under clinical conditions.

In conclusion, we present one of the first and one of the largest cohorts to establish population-based reference values for traditional HRV measures and for advanced measures of cardiac autonomic function. The markers DC, quantifying cardiac parasympathetic tone, and PRD, quantifying cardiac sympathetic tone, have not previously been described and quantified in the population. We demonstrate that traditional HRV measures and advanced measures of cardiac autonomic function are largely independent of sex. PRD is a marker that is particularly stable across age and is not relevantly modified in the context of cardiovascular conditions. It will be a future research questions to test, whether our tested measures including DC and PRD are predictive of clinical outcomes in the general population.
